# Clinical outcomes of an unplanned second debridement, antibiotics and implant retention (DAIR) procedure in acute postoperative prosthetic joint infections

**DOI:** 10.1007/s00264-025-06617-x

**Published:** 2025-08-05

**Authors:** Juan Carlos Perdomo-Lizárraga, Andrés Combalia, Jenaro A. Fernández-Valencia, Juan Carlos Martínez-Pastor, Alfonso Alías, Laura Morata, Álex Soriano, Ernesto Muñoz-Mahamud

**Affiliations:** 1 Department of Orthopaedics and Trauma Surgery, Hospital Clínic of Barcelona. University of Barcelona. Barcelona, Spain., Barcelona, Spain; 2 Departament de Cirurgia i Especialitats Medicoquirúrgiques, Facultat de Medicina i Ciències de la Salut, Universitat de Barcelona (UB), c. Casanova, 143, 08036, Barcelona, Spain; 3 August Pi i Sunyer Biomedical Research Institute, Barcelona, Spain; 4 Department of Infectious Diseases, Hospital Clínic of Barcelona. University of Barcelona. Barcelona, Spain, Barcelona, Spain; 5 CIBERINF, Ciber in Infectious Diseases, ISCIII, Madrid, Spain, Barcelona, Spain

**Keywords:** Prosthetic joint infection, Debridement, Hip arthroplasty, Knee arthroplasty

## Abstract

**Introduction:**

Debridement, antibiotics, and implant retention (DAIR) is a commonly employed strategy for managing acute postoperative prosthetic joint infections (PJI) while preserving the prosthesis. However, the clinical value of an unplanned second DAIR - performed due to inadequate infection control - remains controversial and is often considered a potential treatment failure. This study aimed to compare the two year clinical outcomes of patients undergoing a single DAIR versus those requiring an unplanned second DAIR for acute postoperative PJI of the hip or knee.

**Methods:**

We retrospectively reviewed electronic medical records of patients treated with DAIR for acute postoperative PJI between January 1999 and December 2020. Patients were categorized into two groups: those managed with a single DAIR (DAIR-1 group) and those requiring an unplanned second DAIR within 12 weeks (DAIR-2 group). Treatment failure was defined as any of the following: further debridement beyond 12 weeks, revision surgery with prosthesis removal, initiation of long-term suppressive antibiotic therapy, or PJI-related mortality. Patients lost to follow-up before two years were excluded.

**Results:**

A total of 318 patients were included, with 292 in the DAIR-1 group and 26 in the DAIR-2 group. Mean follow-up was 89.4 months. At two years, revision surgery was required in 19.2% (56/292) of DAIR-1 patients and 42.3% (11/26) of DAIR-2 patients (*p* = 0.005). Overall failure-free survival at two years was observed in 75.3% (220/292) of DAIR-1 patients compared to 46.2% (12/26) of those in the DAIR-2 group (*p* = 0.001).

**Conclusion:**

Unplanned second DAIR procedures are associated with significantly lower success rates at two years. Nonetheless, given that nearly half of these patients remained free of failure, a second DAIR may still be a reasonable therapeutic option in selected cases, provided that the increased risk of a poorer prognosis is taken into account.

## Introduction

The incidence of prosthetic joint infection (PJI) ranges from 0.5 to 2% in knee arthroplasties and from 0.5 to 1% in primary hip arthroplasties [1]. In revision surgeries, this incidence increases significantly, reaching 3– 10% [2]. Debridement, antibiotic therapy, and implant retention (DAIR) is the most frequently recommended strategy for managing acute PJI, offering important advantages such as implant preservation, bone stock conservation, and lower resource utilization compared to implant removal procedures [3, 4].

A recent position paper from the European Bone and Joint Infection Society (EBJIS) has provided updated recommendations regarding DAIR as a curative strategy for acute periprosthetic hip and knee infections. The authors specifically state that a DAIR procedure may be considered in patients up to 12 weeks after arthroplasty, provided that symptom duration does not exceed three weeks. However, they also caution that success rates may be lower in such cases [5].

Several factors have been associated with increased risk of DAIR failure, including failure to exchange mobile components during debridement, infections caused by *Staphylococcus aureus* (regardless of methicillin susceptibility) [6–9], and the use of antibiotics lacking anti-biofilm activity [10, 11]. Reported success rates for DAIR vary widely, ranging from 16 to 88% [12].

When infection control is not achieved following an initial DAIR, some patients may undergo an unplanned second DAIR in an attempt to retain the implant and avoid more invasive revision surgery. Although this approach is sometimes used in clinical practice, its rationale and effectiveness remain controversial. A second DAIR may provide an additional opportunity for surgical debridement and targeted antibiotic delivery; However, the need for a second DAIR may also reflect underlying factors such as infection with more virulent or antibiotic-resistant organisms, delayed recognition of the infection, or inadequate surgical or antimicrobial management during the initial procedure. These circumstances are commonly associated with a higher risk of treatment failure [13, 14]. While some studies suggest that undergoing a second DAIR is linked to higher failure rates, the evidence across the literature remains inconsistent and inconclusive [7, 9, 15].

The current study hypothesizes that patients requiring an unplanned second DAIR may experience worse outcomes and higher failure rates compared to those successfully treated with a single DAIR. The aim of this study was to compare two year clinical outcomes between patients undergoing a single DAIR and those who required an unplanned second DAIR for acute postoperative PJI of the hip or knee.

## Materials and methods

### Study design

This retrospective, single-center study evaluated the outcomes of the DAIR strategy for the management of acute postoperative PJI following primary or revision hip and knee arthroplasty. The study included patients treated between January 1999 and December 2020 and was approved by the institutional review board (Register No: HCB/2023/0492).

Eligibility criteria for the initial DAIR procedure included patients with postoperative acute PJI, a stable prosthesis, adequate soft tissue condition, and absence of a sinus tract. Acute PJI was defined as clinical signs and symptoms of infection lasting ≤ three weeks and occurring within 12 weeks of the index arthroplasty. Diagnoses were based on the International Consensus Meeting criteria [16]. A minimum follow-up of two years was required for inclusion.

An unplanned second DAIR was defined as a procedure performed within 12 weeks of the initial debridement due to inadequate infection control. Although the institutional standard of care is a single debridement, a second DAIR is considered within this timeframe if clinical signs and symptoms persist or worsen. Beyond 12 weeks, treatment is deemed a failure, and the standard approach is implant removal or initiation of suppressive antibiotic therapy according to institutional protocol.

### Outcome definition

Treatment failure was defined as any of the following events: additional debridement beyond 12 weeks after the initial procedure, revision surgery involving prosthetic component removal, the requirement for long-term suppressive antibiotic therapy, or PJI-related mortality [17]. Relapse was defined as recurrence of infection caused by the same microorganism, while reinfection was defined as infection with a different microorganism compared to the initial DAIR [18]. Patients lost to follow-up before completing the two year observation period were excluded.

### Statistical analysis

Continuous variables were expressed as means with standard deviations (SD), while categorical variables were presented as absolute frequencies and percentages. Group comparisons for continuous variables were performed using Student’s *t*-test, and categorical variables were compared using the Chi-square test. Kaplan–Meier survival analysis was used to evaluate failure-free survival. The cohort was divided into two groups: the DAIR-1 group, comprising patients managed with a single DAIR, and the DAIR-2 group, comprising those who required an unplanned second DAIR within 12 weeks of the initial procedure. Data were collected using Microsoft Excel and analyzed with Jamovi version 2.0.0.0.

## Results

A total of 318 patients met the inclusion criteria and were analyzed. The DAIR-1 group included 292 patients (91.8%), of whom 163 were female and 129 male. The DAIR-2 group comprised 26 patients (8.2%), with 14 females and 12 males. The mean (SD) follow-up duration was 81.3 (57.4) months in the DAIR-1 group and 90.2 (50.8) months in the DAIR-2 group, with no statistically significant difference (*p* = 0.38). The mean (SD) age at the time of surgery was 70.3 (11.2) years in DAIR-1 and 71.4 (9.9) years in DAIR-2 (*p* = 0.64). The mean (SD) time from index arthroplasty surgery to the first DAIR surgery was 4.1 (2.3) weeks in DAIR-1 and 3.7 (2.2) weeks in DAIR-2 (*p* = 0.45).

Intraoperative cultures obtained during debridement revealed monomicrobial infections in 161 patients (55.1%) in the DAIR-1 group and in 23 patients (88.5%) in the DAIR-2 group (*p* ≤ 0.001) (Table 1). Polymicrobial infections were identified in 80 patients (27.4%) in the DAIR-1 group, while no such cases were recorded during the initial DAIR procedure in the DAIR-2 group (*p* = 0.002). Culture-negative infections occurred in 51 patients (17.5%) in the DAIR-1 group and in 3 patients (11.5%) in the DAIR-2 group (*p* = 0.44).

**Table 1:** Demographics of patients according to the number of required debridements

Variable	All patients (*n* = 318, 100%)		DAIR-1 (*n* = 292, 91.8%)		DAIR-2 (*n* = 26, 8.2%)			
	*n*	%	*n*	%		*n*	%	*p*-value
Sex:								
Female	177	55.7	163	55.8		14	53.8	0.84
Male	141	44.3	129	44.2		12	46.2	
**Site of Arthroplasty**:								
Hip	128	40.3	120	41.1		8	30.8	0.30
Knee	190	59.7	172	58.9		18	69.2	
**Index arthroplasty**:								
Primary	250	78.6	231	79.1		19	73.1	0.47
Aseptic revision	68	21.4	61	20.9		7	26.9	
**Cultures from DAIR**:								
Negative	54	17	51	17.5		3	11.5	0.44
Monomicrobial	184	57.9	161	55.1		23	88.5	**< 0.001**
Polymicrobial	80	25.1	80	27.4		0	0	**0.002**
**Revisions at 2 years**								
Chronic infection	55	17.3	46	15.8		9	34.6	**0.01**
Aseptic loosening	7	2.2	5	1.7		2	7.7	**0.04**
Knee instability	2	0.6	2	0.7		0	0	0.67
Hip dislocation	2	0.6	2	0.7		0	0	0.67
Arthrofibrosis	1	0.3	1	0.3		0	0	0.76
Total	67	21.1	56	19.2		11	42.3	**0.005**

The most frequently isolated pathogen in the DAIR-1 group was coagulase-negative staphylococci (CoNS), identified in 101 cases (34.6%). In contrast, *Staphylococcus aureus* was the predominant pathogen in the DAIR-2 group, isolated in 8 patients (30.8%). The full microbiological profile of the isolates is presented in Table 2.

**Table 2:** Summary of microorganisms isolated from intraoperative cultures obtained during the initial debridement

Microorganism	All patients (*n* = 318, 100%)		DAIR-1 (*n* = 292, 91.8%)		DAIR-2 (*n* = 26, 8.2%)	
	*n*	%	*n*	%	*n*	%
Coagulase-negative staphylococci	107	33.6	101	34.6	6	23.1
*Staphylococcus aureus*	84	26.4	76	26	8	30.8
*Pseudomonas aeruginosa*	30	9.4	29	9.9	1	3.8
*Escherichia coli*	30	9.4	27	9.2	3	11.5
*Enterococcus faecalis*	27	8.5	27	9.2	0	0
*Proteus mirabilis*	15	4.7	15	5.1	0	0
*Enterobacter cloacae*	13	4.1	13	4.5	0	0
*Klebsiella pneumoniae*	12	3.8	11	3.8	1	3.8
*Morganella morganii*	5	1.6	4	1.4	1	3.8
*Serratia marcescens*	4	1.3	3	1	1	3.8
*Citrobacter koseri*	3	0.9	3	1	0	0
*Streptococcus agalactiae*	2	0.6	2	0.7	0	0
*Candida parapsilosis*	2	0.6	2	0.7	0	0
*Peptostreptococcus anaerobius*	2	0.6	2	0.7	0	0
Other microorganisms	17	5.3	15	5.1	2	7.7

Among the 26 patients in the DAIR-2 group, 65.4% (17/26) had positive cultures during the second debridement. In seven of these cases (26.9%), the same microorganism was isolated as in the first procedure, while in ten cases (38.5%), a different microorganism was identified. During the second debridement 13 monomicrobial infections (50%) and 4 polymicrobial infections (15.4%) were identified. The mean (SD) time from the first DAIR surgery to the second DAIR surgery was 3.3 (2.6) weeks.

### Follow-up at two years

**Fig. 1:**
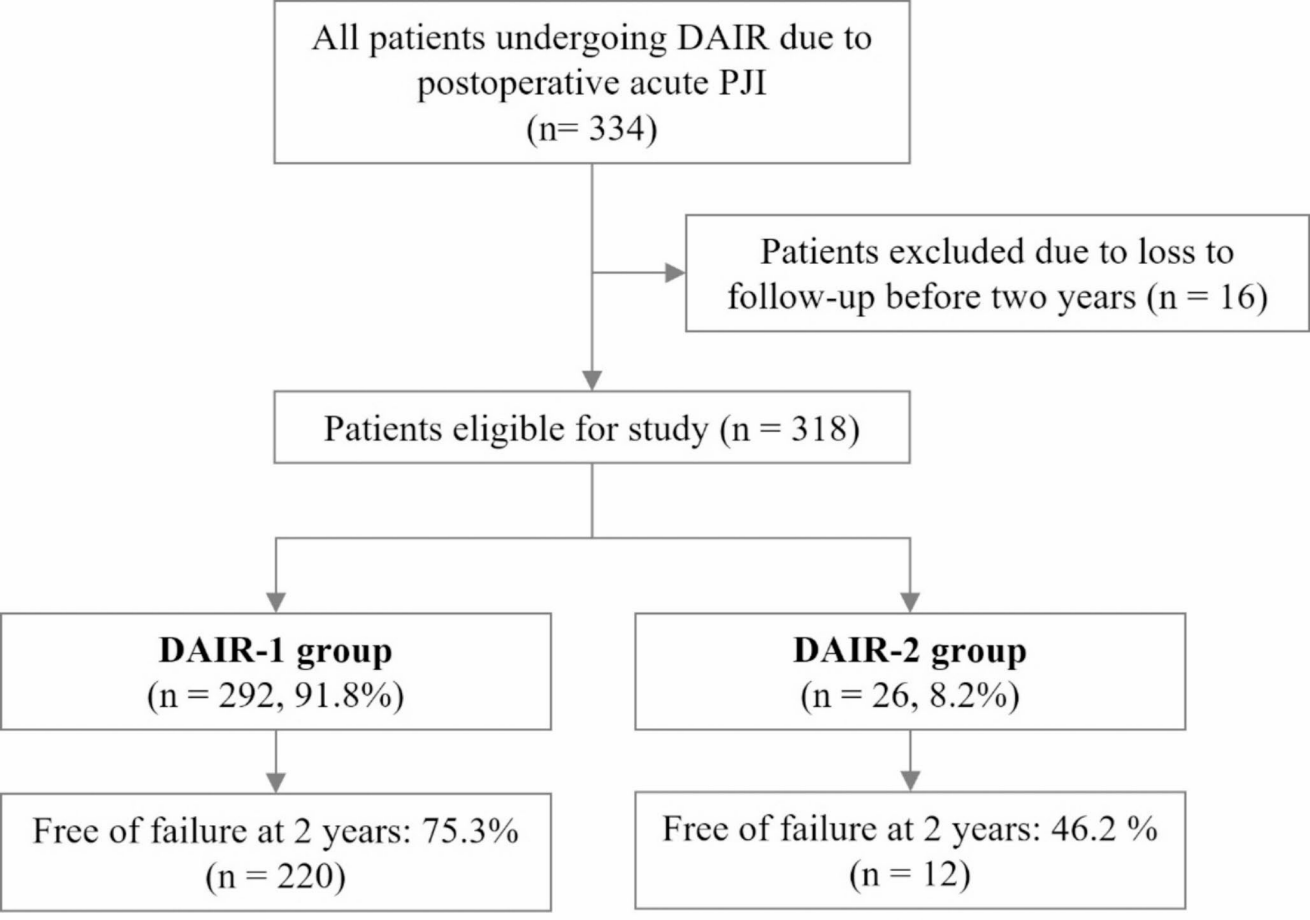
Patient inclusion flowchart. Failure was defined as the need for additional debridement beyond 12 weeks after the initial procedure, revision surgery involving prosthetic component removal, the requirement for suppressive antibiotic therapy, or PJI-related mortality

Failure-free survival at two years was achieved in 220 of 292 patients (75.3%) in the DAIR-1 group and in 12 of 26 patients (46.2%) in the DAIR-2 group (*p* = 0.001). A flowchart illustrating patient selection and study inclusion is shown in Fig. 1. Among patients who died within the two-year follow-up period without implant removal, 11 of 292 (3.8%) in the DAIR-1 group died − two patients (0.7%) due to PJI-related causes and nine (3.1%) due to unrelated causes. In the DAIR-2 group, three patients (11.5%) died of unrelated causes (*p* = 0.03). Additional DAIR beyond 12 weeks without prosthesis removal was required in four patients (1.4%) in DAIR-1 whereas no cases needing additional DAIR were recorded in the DAIR-2 group (*p* = 0.54). Only one patient (0.3%) in the DAIR-1 group required long-term suppressive antibiotic therapy. Details regarding patient failures are presented in Table 3.

**Table 3:** Two-Year outcomes: Failure-Free survival and distribution of treatment failures

Variable	All patients (*n* = 318, 100%)		DAIR-1 (*n* = 292, 91.8%)		DAIR-2 (*n* = 26, 8.2%)		
	*n*	%	*n*	*n*	%	*n*	*p*-value
PJI-related deaths	2	0.6	2	0.7	0	0	0.67
Additional debridement performed after 12 weeks	4	1.3	4	1.4	0	0	0.54
Suppressive antibiotic therapy	1	0.3	1	0.3	0	0	0.76
Revision surgeries	67	21.1	56	19.2	11	42.3	**0.005**
**Total failure at 2 years**	74	23.3	63	21.6	11	42.3	**0.01**
Deaths due to unrelated causes	12	3.8	9	3.1	3	11.5	**0.03**
**Failure-free survival at 2 years**	232	73	220	75.3	12	46.2	**0.001**

**Fig. 2:**
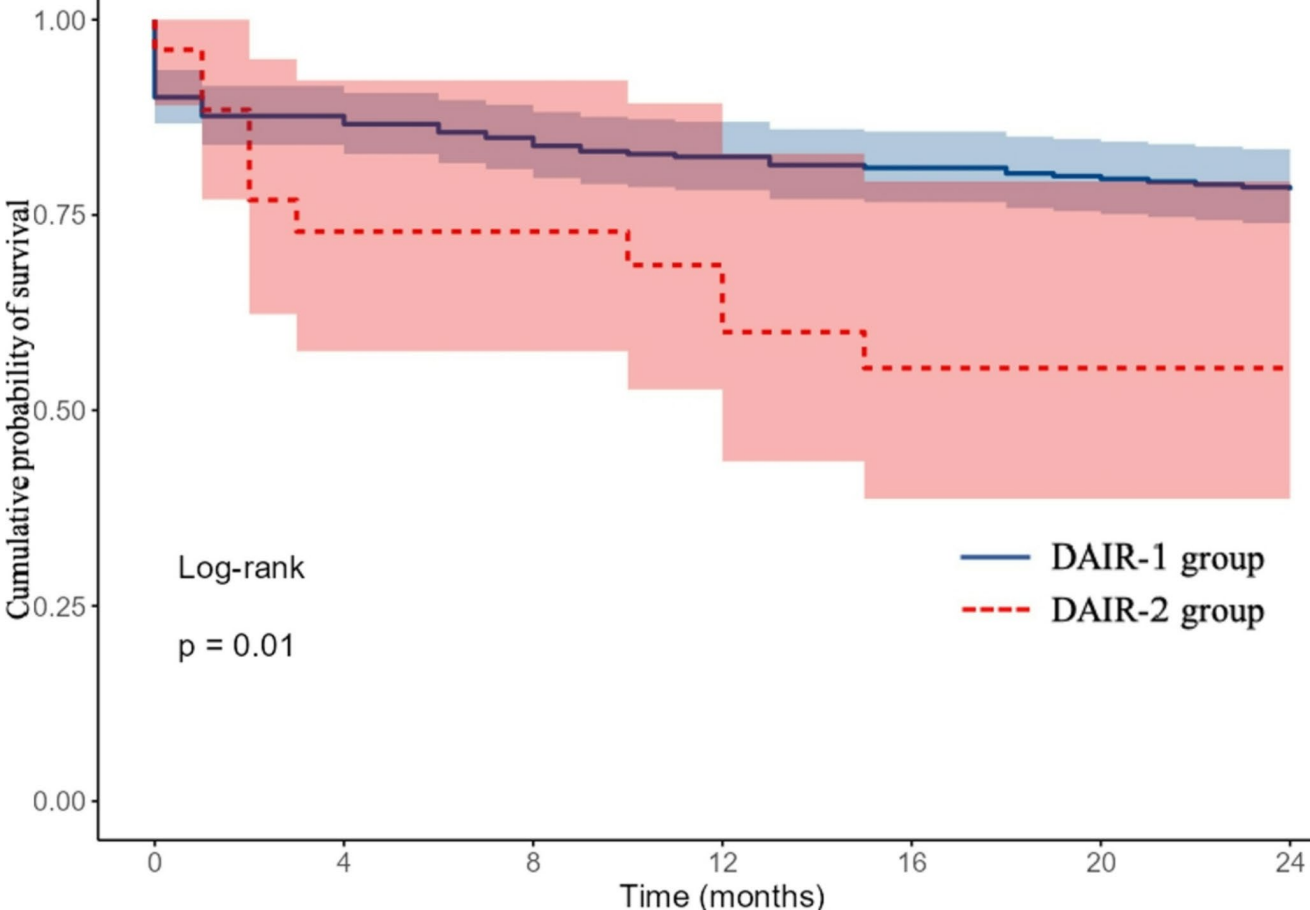
Kaplan–Meier survival curve showing the cumulative probability of failure-free survival over a 24-month follow-up period (Y-axis). The solid blue line represents patients who underwent a single DAIR (DAIR-1 group), while the red dashed line corresponds to those who required an unplanned second DAIR (DAIR-2 group). The shaded area indicates the 95% confidence interval (CI)

The two year all-cause revision rate was significantly higher in the DAIR-2 group (42.3%, 11/26) than in DAIR-1 (19.2%, 56/292; *p* = 0.005). Among those revised, intraoperative cultures were positive in 53.6% (30/56) of DAIR-1 cases and 72.7% (8/11) of DAIR-2 cases (*p* = 0.24). The relapse rate among revised cases was 30.4% (17/56) in DAIR-1 and 27.3% (3/11) in DAIR-2 (*p* = 0.83). Reinfections were observed in 23.2% (13/56) of DAIR-1 and 45.5% (5/11) of DAIR-2 patients (*p* = 0.12). At 24 months, prosthesis survival was 79% in the DAIR-1 group and 55% in the DAIR-2 group. The cumulative failure-free survival probability at two years is illustrated in the Kaplan–Meier curve (Fig. 2).

In hip infections, failure occurred in 24.2% of DAIR-1 patients and 50% of DAIR-2 (*p* = 0.10), with prosthesis survival rates of 76% and 47%, respectively. In knees, failure rates were 20.3% in DAIR-1 and 38.9% in DAIR-2 (*p* = 0.07), with corresponding survival of 81% and 59%. While no direct statistical comparison was performed between joint types, the data suggest a non-significant trend toward better outcomes in knees.

## Discussion

DAIR is an effective strategy in selected patients, with implant survival rates exceeding 80% at five years [19, 20]. Nonetheless, prosthetic component removal is recommended when the initial DAIR fails [21]. In contrast, EBJIS guidelines [5] suggest that a second DAIR may be considered when the implant is stable, soft tissues are intact, and the first procedure was incomplete - particularly if mobile components were not exchanged. However, when signs of persistent infection are present, implant removal should be prioritized if the goal is infection eradication.

In our cohort, 8.2% of patients underwent an unplanned second DAIR, with significantly higher failure rates than those managed with a single procedure. Compared to the study by Wouthuyzen-Bakker et al. [17], where 31.7% underwent a second DAIR with a lower revision rate (16%), our stricter selection criteria may explain both the lower rate of second procedures and the higher failure rate (42.3%, 11/26). Additionally, the lower proportion of culture-negative infections in our series (34.6%, 9/26) vs. 62% reported by them, suggests more targeted decision-making. While a more permissive approach may benefit some patients, it risks overtreatment in borderline cases.

Our failure rates (21.6% for single DAIR, 42.3% for second DAIR) were lower than those reported by Triantafyllopoulos et al. [22] and slightly better than the 68% cure rate reported by Veerman et al. [15]. These differences may reflect variations in surgical protocols and patient selection. A second DAIR yielded success in 54.5% of patients in the multicenter study by Auñón et al. [23], where failure was linked to factors such as lack of modular component exchange and non-specialist teams, reinforcing the importance of optimized surgical and antimicrobial strategies. Replacing mobile components has been consistently associated with higher DAIR success [8, 24–26]. Zhu et al. [8] reported nearly twice the success rate when components were exchanged. In this context, a second DAIR may be justified in selected patients - particularly those without component exchange during the initial procedure or high surgical risk. However, the elevated failure risk must be carefully weighed.

While the optimal timing for DAIR remains a subject of ongoing debate, a recent study from our group found no significant differences in outcomes between procedures performed within the first four weeks and those conducted between weeks five and twelve [27]. In the present study, the mean interval between the index arthroplasty and the initial DAIR procedure was not significantly different between patients who underwent a single DAIR and those who eventually required a second DAIR.

The main limitation of this study is its retrospective design, which carries an inherent risk of selection and information bias. In particular, among patients who underwent a second DAIR procedure, it was not consistently possible to determine whether mobile components had been exchanged during the initial surgery or whether procedures were performed electively or by on-call surgical teams. Additionally, the DAIR-2 group was relatively small (*n* = 26), which may limit the statistical power of some subgroup comparisons. The absence of a true control group and potential confounders not accounted for in the analysis also restrict the interpretation of causal relationships. Despite these limitations, the 21-year study period and consistent diagnostic criteria provide valuable insight into the outcomes of this treatment strategy in a real-world clinical setting.

## Conclusion

Compared to a single procedure, an unplanned second DAIR was associated with lower failure-free survival at two years (75.3% vs. 46.2%) and higher revision rates (19.2% vs. 42.3%). Despite these differences, 46.2% of patients treated with a second DAIR remained free of failure at two years. Therefore, a second DAIR may still be a reasonable option in selected cases, provided the elevated risk of failure is clearly acknowledged and discussed with the patient.

## Data Availability

No datasets were generated or analysed during the current study.
